# Biodiversity of Yeast Species Isolated During Spontaneous Fermentation: Influence of Grape Origin, Vinification Conditions, and Year of Study

**DOI:** 10.3390/microorganisms13071707

**Published:** 2025-07-21

**Authors:** Ana Benito-Castellanos, Beatriz Larreina, María Teresa Calvo de La Banda, Pilar Santamaría, Lucía González-Arenzana, Ana Rosa Gutiérrez

**Affiliations:** 1 ICVV, Instituto de Ciencias de la Vid y el Vino (Universidad de La Rioja, Gobierno de La Rioja, CSIC), Finca La Grajera, Ctra. LO-20-Salida 13, 26071 Logroño, La Rioja, Spain; ana.benito@icvv.es (A.B.-C.); beatriz.larreina@icvv.es (B.L.); psantamaria@larioja.org (P.S.); 2 Bodegas Bilbaínas, Calle Estación s/n, 26200 Haro, La Rioja, Spain; m.calvo@bodegasbilbainas.com

**Keywords:** spontaneous fermentation, yeasts species, wine, biodiversity, microbial terroir

## Abstract

Winemaking involves a microbial ecosystem where yeast diversity, shaped by terroir and winemaking conditions, determines wine characteristics. Understanding the microbial diversity of vineyards and spontaneous fermentation is crucial for explaining a winery’s typical wine profile. Studying and inoculating indigenous strains make it possible to produce high quality wines, reflecting the production environment. This study analyzes the yeast species involved in 16 spontaneous fermentations (8 in 2022 and 8 in 2023) from grapes of four distinct vineyards under two sets of winemaking conditions. A total of 1100 yeast colonies were identified by MALDI-TOF and DNA sequencing techniques. *Saccharomyces* (*S.*) *cerevisiae* and *Hanseniaspora uvarum* were the most prevalent species, alongside significant populations of non-*Saccharomyces* yeasts such as *Lachancea thermotolerans* and *Metchnikowia pulcherrima*, which were the most abundant ones. Minor yeast species, including *Aureobasidium pullulans*, *Starmerella bacillaris*, *Kazachstania servazzi*, and other *Hanseniaspora* spp., were also detected. The results demonstrated that yeast diversity in spontaneous fermentations varied according to vineyard origin and winemaking conditions. Differences between the two vintages studied indicated that annual climatic conditions significantly influenced yeast diversity, especially among non-*Saccharomyces* species. This substantial diversity represents a valuable source of indigenous yeasts for preserving the typicity of a winery’s wines under controlled conditions.

## 1. Introduction

Spontaneous fermentation, the most traditional and minimally interventive approach to alcoholic fermentation, yields wines of notable complexity. This complexity arises from the intricate interplay of diverse yeast species, encompassing both *Saccharomyces* and non-*Saccharomyces* varieties [1]. However, the inherent variability of indigenous microbiota renders this method unpredictable. Consequently, the modern wine industry has widely adopted inoculation with *S. cerevisiae* to mitigate these risks. Nonetheless, recent research has demonstrated that monoculture fermentations using highly efficient *S. cerevisiae* strains fail to replicate the complexity achieved through diverse yeast populations. This diversity, conversely, enhances complexity and allows winemakers to express distinct regional or varietal characteristics, thereby enhancing brand differentiation in a competitive market [2]. Furthermore, non-*Saccharomyces* yeasts are now recognized not as disruptive agents, but as valuable biotechnological tools for modulating wine attributes [3,4,5,6,7]. Accordingly, commercial non-*Saccharomyces* strains are increasingly employed in sequential inoculations with *S. cerevisiae* to refine the physicochemical and sensory profiles of wines [8].

While starter cultures have improved the reproducibility and predictability of wine quality, concerns persist regarding the homogenization of wine styles due to the widespread use of commercial strains. This practice diminishes the diversity of indigenous yeasts, particularly non-*Saccharomyces* species, thereby compromising regional typicity and distinct producer styles. In response to growing consumer demand for innovative wines and market competition, there is an increasing emphasis on wine typicity linked to specific geographical areas (terroir), wineries, or single vineyards. The concept of microbial terroir, which recognizes the influence of indigenous yeast composition on grape characteristics and final wine attributes, has gained prominence [9,10,11]. The massive use of commercial yeast has reduced the potential contributions of native yeast populations (*Saccharomyces* and non-*Saccharomyces*) [2]. In addition, regional dispersion of native yeast cells has been estimated to cover an area not more than 10 km away from their origin [12]. This ensures a restricted limit for microbiota diversity within a few vineyards of a certain microregion, mainly for non-*Saccharomyces* strains [13]. Thus, inoculation with indigenous yeasts from specific vineyards offers a strategy to produce wines with unique and distinctive styles.

Moreover, the burgeoning interest in organic wines has prompted winemakers to explore additive-free winemaking, notably reducing or eliminating sulfites. This approach alters yeast ecology and fermentation dynamics, potentially impacting wine quality. Harnessing natural biodiversity and implementing biocontrol agents to optimize winemaking management can facilitate the production of typical, high-quality wines [14,15].

Consequently, many wineries are implementing programs to isolate and select indigenous yeasts, both *Saccharomyces* and non-*Saccharomyces*, based on their representation of the local terroir. These selections are intended for use as inocula, ensuring the typicity, complexity, and stability of wines. Employing native yeast selections is a crucial strategy for securing quality initial fermentation and mitigating the risks associated with uncontrolled spontaneous fermentation. Prior to selection, ecological and biodiversity studies of yeast populations during spontaneous alcoholic fermentation are essential for understanding the complex interactive networks that govern species dominance. This research aims to investigate the yeast ecology of spontaneous fermentations in a commercial winery over two consecutive vintages. The study focuses on analyzing the diversity of yeast populations in relation to grape vineyard origin, winemaking conditions, and vintage variations.

## 2. Materials and Methods

### 2.1. Grapes and Spontaneous Fermentation

In this study, conducted over two consecutive vintages (2022 and 2023), grapes of two red varieties (Tempranillo and Graciano) from four vineyards (1, 2, 3, and 4), located in La Rioja (Spain) and belonging to Bodegas Bilbaínas, were used. These vineyards are located within a radius of less than 10 km of each other. Vineyards 2 and 3 are located in the same plantation, but vineyard 2 is made up of Tempranillo grapes and vineyard 3 of Graciano grapes. These vineyards have yielded grapes for the winery’s premium wines over recent decades. For this reason, they have set out to study the microbiota present in the spontaneous fermentations carried out with these grapes, with the objective of developing proprietary yeast inoculums.

Grapes were manually harvested at optimal ripeness (26 September 2022, and 29 September 2023). For each grape batch, two distinct spontaneous fermentation protocols were implemented: laboratory fermentation (LF), conducted in sterile low-capacity bags (without sulfur dioxide addition or aeration and at a temperature of 23 °C in a refrigerated environment) and winery fermentation (WF), performed under industrial-scale conditions (sulfites addition: 60 mg SO_2_/kg of grapes, pump-overs, and temperature control at 28 °C). LF involved aseptically processing 2 kg of grapes from each sample at harvest. Grapes were crushed within sterile 3 L bags to initiate spontaneous fermentation, relying solely on the indigenous yeast populations present on the grapes. WF, utilizing the remaining grapes from each vineyard plot, were conducted at the winery’s facilities in 1500 L wine tanks under standard commercial conditions.

### 2.2. Yeast Isolation and Identification

For LF, yeast isolation samples were collected at specific time points: 48 h and 96 h seven and ten days post-vatting in 2022. In 2023, the initial sampling schedule was modified, with the 48 and 96 h samples replaced by a single 72 h sample. In WF, must samples were collected at 72 h post-vatting and during tumultuous fermentation, defined by a must density of 1.025. All samples were collected in sterile 50 mL Falcon tubes and transported to the laboratory for subsequent analysis.

The must samples were processed according to the method of successive decimal dilutions and sowing in Petri dishes containing the appropriate culture media: GYP (20 g glucose, 20 g agar, 5 g mycological peptone, and 5 g yeast extract in 1 L of water) (Scharlau, Barcelona, Spain) and Agar-Lysine (66 g Lysine medium in 1 L of water with 10 mL of 50% potassium lactate and 1 mL of 2.5% bromocresol green dissolved in absolute ethanol) (Scharlau, Barcelona, Spain). GYP medium facilitated the growth of a broad range of yeast species, while Agar-Lysine medium selectively promoted growth yeasts with the ability to utilize lysine as a nitrogen source. Plates were incubated at 28 °C for 48 h, and colonies were then randomly isolated from plates containing between 30 and 300 colonies (15 colonies from the Agar-Lysine medium and 10 from the GYP medium). A total of 1100 yeast colonies were isolated: 600 from the 2022 vintage and 500 from the 2023 vintage. The isolation medium was not differentiated in the subsequent analysis of these 25-colony sets.

Yeast isolates were identified to the species level using Matrix-Assisted Laser Desorption/Ionization-Time of Flight (MALDI-TOF) mass spectrometry (Bruker Daltonik GmbH, Bremen, Germany). This technique, which has been validated for microorganisms of oenological origin [16], produces a characteristic and reproducible protein spectrum that can be used to differentiate microorganisms at genus and species level. Mass spectra were acquired using a Microflex Mass Spectrometer (Bruker Daltonik GmbH) equipped with a nitrogen laser (λ = 337 nm), controlled by flexControl software (Version 3.4; Bruker Daltonik GmbH, Bremen, Germany). For identification, a fresh yeast culture was placed directly on a target plate using a sterile toothpick. An on-target extraction was then carried out by applying 1 µL of formic acid directly onto the spotted microorganism to increase cell lysis. Once dried, the spotted microorganism was overlaid with a matrix solution (HCCA). Once the matrix was dry, the target plate was loaded into the ionization chamber, where the ionized molecules are accelerated by an electric charge and travel through a vacuum tube to a detector. On their way, the ionized molecules are separated according to their mass-to-charge ratio [17]. The instrument’s mass analyzer measures the time required for each ion to reach the detector and records the TOF (time of fly), generating a mass spectrum. The spectrum was then compared to a reference spectrum database (MALDI Biotyper) to identify the unknown microorganism. A score ≥ 2.0 indicates high confidence identification; scores between 1.70 and 1.99 indicate low confidence identification; and scores ≤ 1.70 indicate that it is not possible to identify the organism.

Isolates that yielded inconclusive MALDI-TOF profiles were subjected to sequencing. DNA was extracted from fresh cultures using the rapid extraction protocol without the Taq polymerase inhibitors described by López [18]. Specifically, 250 µL of lysis buffer (Tris 50 mM, pH 8; β-mercaptoethanol 10 mM) from a pure culture on a plate, grown for 48 h, was collected with an inoculation loop and resuspended in an Eppendorf tube. This mixture was vortexed, allowed to stand for 10 min at room temperature, and then subjected to a 100 °C water bath for 10 min in boiling water. While still hot, it was vortexed again and centrifuged at 13,000 rpm for 3 min. The supernatant, containing the genomic DNA, was collected in a sterile Eppendorf tube and 10 µL of this was used for each via polymerase chain reaction (PCR).

The D1/D2 domain was amplified by PCR using the primers and conditions described by Kurtzman and Robnett [19]. PCR products were purified and sequenced by Macrogen Spain to determine the yeast genus and species. The resulting sequences were used for comparison to the GenBank database using the Basic Local Alignment Search Tool (BLAST) in the NCBI database (https://blast.ncbi.nlm.nih.gov/Blast.cgi accessed on November 2024) to determine the species identity of each isolate.

## 3. Results and Discussion

### 3.1. Grape Must Maturity and Climatic Influences

The characteristics of the four batches of grapes used in the two years are shown in Table 1. Within each vintage, variations in probable alcoholic strength were observed across vineyards, despite their proximity (<10 km) and thus similar regional climate. These variations underscore the influence of vineyard-specific attributes (location, orientation, variety, and soil type) on grape composition during ripening. Furthermore, significant inter-annual climatic variations, as reported by regional meteorological services [20], impacted grape maturity. The 2022 vintage was characterized by warmer and drier conditions, including heatwaves and minimal rainfall, compared to the more moderate 2023 vintage with cooler nights and regular precipitation. As anticipated, vineyards 2 and 3 exhibited higher probable alcohol content in 2022. However, vineyard 1 showed slightly lower ripeness in 2022, and vineyard 4 showed minimal inter-annual differences. These discrepancies highlight the differential impact of climatic conditions on vineyards with varying characteristics, consistent with the observation that vineyard adaptation to climate change varies [21,22].

### 3.2. Distribution of Yeast Species in the Different Stages of Fermentation

**Figure 1:**
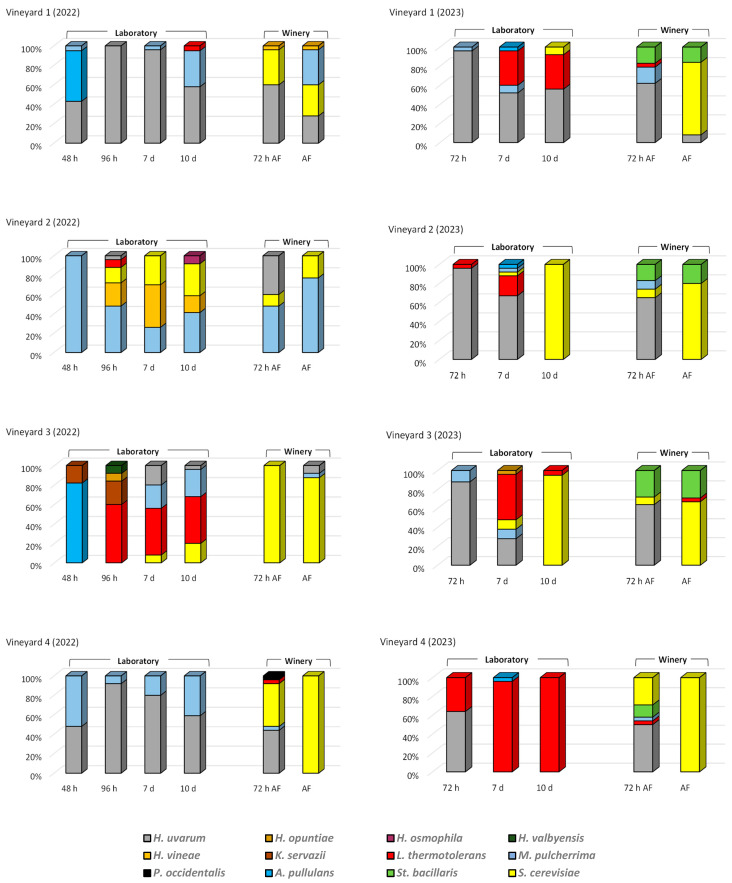
Yeast species (%) present at different times during fermentations carried out on grapes from 4 vineyards under different conditions (laboratory and winery) in two consecutive years (2022 and 2023). A significant abundance of non-*Saccharomyces* yeasts was observed during early fermentation, followed by an increase in *S. cerevisiae* abundance (particularly in WF) as fermentation progressed, as has been described by other authors [24]. However, in LF, *S. cerevisiae* dominance was less pronounced, with absence in three out of eight fermentations. These observations align with findings by Shimizu et al. [25] in non-sulfited sterile fermentations. The limited *S. cerevisiae* presence in early LF stages reflects its low abundance on grape surfaces. The prevalence of non-*Saccharomyces* yeasts in LF is likely attributable to low temperatures and the absence of sulfur dioxide [26]. These results suggest that bioprotection strategies, involving early inoculation with fermentative yeasts, are crucial for stabilizing non-sulfited fermentations [15].

Figure 1 illustrates the relative abundances of yeast species during laboratory fermentation (LF) and winery fermentation (WF) across four vineyards over 2022 and 2023. Consistent with the expectations presented by Portillo and Mas [23], who showed that the genera *Hanseniaspora* and *Candida* were dominant during initial and mid fermentation while the final fermentation was mainly dominated by *Candida* and *Saccharomyces*, a temporal shift in microbial populations was observed, with *H. uvarum* dominating early fermentation stages. However, exceptions were noted in LF from vineyards 2 and 3 (2022), where *H. uvarum* was minimally detected.

In 2023, the *S. cerevisiae* species dominated fermentation progress in almost all WFs. However, in 2022, tumultuous fermentation of vineyards 1 and 2 exhibited high abundances of non-*Saccharomyces* yeasts, particularly *Metchnikowia* (*M.*) *pulcherrima.* This species is recommended in winemaking for its potential to reduce ethanol content, enhance aroma, and provide biocontrol due to its antimicrobial properties against spoilage yeasts [27].

Together with *M. pulcherrima*, *Lanchancea* (*L.*) *thermotolerans* was another prevalent species that contributes to increased acidity and reduced ethanol production, mitigating climate change impacts while also influencing the aromatic profile, which is one of the most important characteristics determining the wine’s distinctive character [28]. This species has been detected mainly in the LF of vineyard 3 in 2022 and in all LFs in 2023, either occasionally or in all phases of LF.

Among the minor non-*Saccharomyces* yeasts detected, *Starmerella* (*St.*) *bacillaris* (synonym *Candida zemplinina*) was isolated in all WF, but only in 2023. Furthermore, the species *K. servazzii* and several species of *Hanseniaspora* (*H.*) (*H. opuntiae*, *H. osmophila*, and *H. vinae*), were detected only in 2022. *St. bacillaris* is a cryotolerant and osmotolerant species present in oenological environments that used together with *S. cerevisiae* enhances the analytical composition and aroma profile of wine [29]. *Kazachstania* (*K.*) *servazzi* is a less studied and less common yeast in winemaking. It has been found on grape surfaces or grape must in different countries, but with low frequency. However, it has been shown to have similar fermentative lifestyles to *S. cerevisiae* and to provide positive aromatic attributes in wine that should be further explored [30].

Although the genus *Hanseniaspora* has always been associated with spoilage during winemaking, recent studies have re-evaluated the role of various species of this genus in wine production mainly as partners of *S. cerevisiae* in fermentations. *H. uvarum*, the most abundant species of this group and traditionally considered detrimental to wine quality, has been described recently as suitable and beneficial for winemaking due to its ability to increase wine complexity [31,32]. Badura et al. [33] observed lactic acid depletion in fermentations with *H. osmophila* and the increase of certain terpenols with *H. opuntiae*. One species in particular, *H. vineae*, has been repeatedly shown to provide several desirable aroma compounds during fermentation, up to 50 orders more than *S. cerevisiae* [34], which led to the development of a commercially available starter culture.

### 3.3. Influence of Winemaking Conditions (LF and WF) on Yeast Species Distribution

Important differences in yeast species distribution were observed between LF and WF, indicating a strong influence of winemaking conditions (laboratory and winery). These differences are evident in the eight grape samples studied. In general, WF exhibited higher *S. cerevisiae* abundances, while LF favored non-*Saccharomyces* species. In the WF there was a similar distribution in the eight vinifications: majority presence of non-*Saccharomyces* yeasts at 72 h and of *S. cerevisiae* in the tumultuous fermentation. The lower competitiveness of non-*Saccharomyces* yeasts in WF is attributed to their lower stress tolerance [35] so that their participation in WF was lower.

These differences between LF and WF are likely due to variations in fermentation conditions. WF involves larger volumes, sulfite addition to the crushed grapes, temperature control at 28 °C, and daily pump-overs during the winemaking process, whereas LF involved smaller volume, no sulfites, lower temperatures (23 °C), and no aeration. Fermentations conducted at low temperature may show a greater contribution of non-*Saccharomyces* species [36] and, in addition, these species show higher sensitivity to SO_2_ [37]. Oxygen availability also impacts yeast survival and dominance [38,39]. All of this resulted in yeasts with little or no fermentative power and that are more sensitive to SO_2_ being more abundant in the LF. *L. thermotolerans* and *M. pulcherrima*, along with *H. uvarum*, were the predominant isolated non-*Saccharomyces* species. Within these two species, *L. thermotolerans* was isolated almost exclusively from LF, while *M. pulcherrima* was present in both LF and WF. In addition, *St. bacillaris* was only isolated from all WF in 2023 and was not detected in any of the LF. So, the conditions under which fermentations are carried out influence the different adaptations of the yeasts that drive the fermentations in the cellar.

Inoculation with winery-resident *S. cerevisiae* strains [40,41,42] may also contribute to differences between LF and WF. *S. cerevisiae* is rarely found on grape surfaces [43] suggesting that its presence in spontaneous fermentations is primarily due to winery environments. The dominance of non-*Saccharomyces* during sterile spontaneous fermentation of grapes (LF), even in some cases where they exclusively managed the fermentation during the 10 days, highlights the impact of winemaking conditions. Only in the LF of plots 2 and 3 in 2023, in the sample taken after 10 days, did the *S. cerevisiae* species dominate, while in the rest of the 2023 and in all the 2022 LF, this phase of fermentation was led by non-*Saccharomyces* yeasts. Thus, *S. cerevisiae* represented a small contribution to the overall broad species diversity found in the LFs of this study. However, the presence of *S. cerevisiae* in some LF samples indicates its potential as a source of vineyard-specific strains. Shimizu et al. [25] also detected this yeast species in laboratory-scale spontaneous winemaking using sterilized labware to avoid winery-resident microbes.

### 3.4. Influence of Grape Origin and Vintage on Yeast Species Distribution

Significant differences were observed in the yeast species present in the fermentations depending on the origin of the grapes. In LFs, where only grape yeasts were involved, the differences between yeast species present in fermentations with grapes from different vineyards each year were greater than in WFs, which would indicate a clear vineyard effect. Tempère et al. [44] indicated that vineyard management and soil and site characteristics influence the grape microbiota that enter a winery. Thus, in 2022 in LFs of grapes from vineyards 1 and 4 *H. uvarum* was the majority species with a slight presence of *M. pulcherrima*, while in the LFs of grapes from vineyards 2 and 3, *M. pulcherrima* and *L. thermotolerans*, respectively, were the majority species. In addition, only a small presence of *S. cerevisiae* was detected in the fermentations of vineyards 2 and 3. However, in 2023 LFs, *S. cerevisiae* was prevalent in vineyards 2 and 3, while *L. thermotolerans* dominated in vineyards 1 and 4.

The grape variety in vineyard 3 was Graciano and in the other three vineyards it was Tempranillo. Although in 2022 the differences between the microbiota in LFs of vineyard 3 and the rest were evident, in 2023 the distribution of species was similar, and in this year the greatest differences occurred in LFs of vineyard 4. Therefore, contrary to what was indicated by other authors [9], it seems that the variety alone is not a determining factor in the development of yeasts on the grapes. It should be noted that vineyards 2 and 3 are within the same plantation, and there were differences between them, especially in 2022, which would indicate that the climatic conditions of each year are much more influential on the microbiota than the grape variety.

Furthermore, in LF, great differences in yeast species were detected in the two years (2022 and 2023) carried out with grapes from the same vineyard, which would indicate a vintage effect. Thus, in vineyards 1 and 4, in both 2022 and 2023, the majority species was *H. uvarum*, and a slight presence of *M. pulcherrima* was detected in 2022 and of *L. thermotolerans* in 2023. In vineyards 2 and 3 in 2022 *H. uvarum* was barely detected and its presence was high in 2023. The main species accompanying *H. uvarum* in 2023 were *L. thermotolerans* and *S. cerevisiae* in both vineyards. In 2022, in fermentations in vineyard 2 the main species were *H. vineae*, *M. pulcherrima* and *S. cerevisiae*, and in vineyard 3 *L. thermotolerans*, *M. pulcherrima* and *K. servazzii*. The differences in the yeasts present in the LF between the two years studied with grapes from the same vineyard were probably due to the different environmental conditions that occurred during the ripening period in the two years of study. Gilbert et al. [9] indicated that vintage can influence microbiological patterns at the level of individual plots, causing noticeable changes in the population. Thus, variations in climatic conditions between years can affect the grape microbiota of a specific vineyard, which can have an impact on the characteristics of the wines obtained.

In the WFs, the distribution of species in the fermentations of vineyards 1 and 4 hardly differed between years 2022 and 2023, while in vineyards 2 and 3 the distribution was very different. Thus, in WF the vineyard origin had a less pronounced effect, with *S. cerevisiae* dominance in tumultuous fermentation likely due to winemaking conditions and winery ecosystem inoculation.

No specific yeast species associated with the vineyard of origin were detected in either LF or WF. However, they were detected depending on the winemaking conditions and the year of study. Some species such as *K. servazzii* were only detected in LF in 2022, others such as *St. bacillaris* only in WF in 2023, and even *L. thermotolerans* were mainly isolated in LF and their presence was sporadic and in a minority in WF.

**Figure 2:**
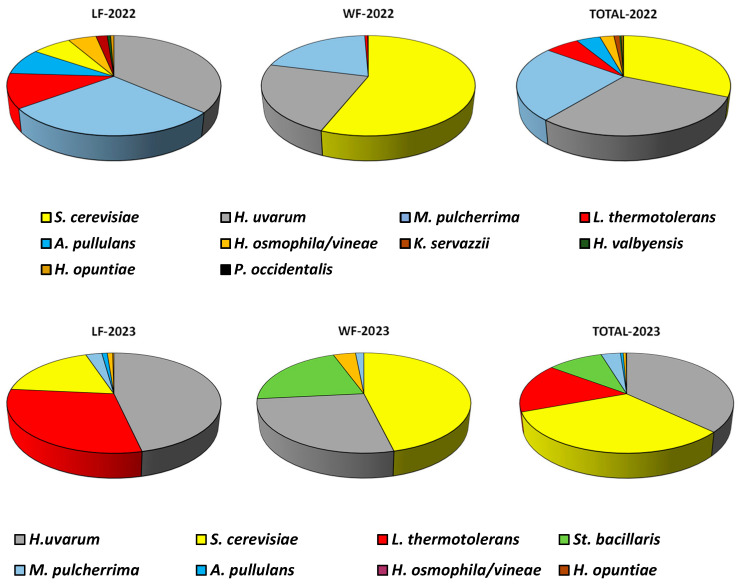
Overall distribution (%) of all the yeast species isolated each year in the 4 laboratory fermentations (LFs), in the 4 cellar fermentations (WFs), and in the total of the 8 laboratory and cellar fermentations carried out for each vintage (total).

There is significant variability in yeast distribution during fermentation, both in grapes from the same vineyards in different years and globally considering all the isolates made each year together (Figure 2). We can see how both *S. cerevisiae* and *H. uvarum* are the species most frequently found and at similar levels in both 2022 and 2023. In both years, colonies of *M. pulcherrima* and *L. thermotolerans* were detected, with the first being more abundant in 2022 and the second in 2023, which could be due to the different weather in each year. We also detected the presence of minority yeasts that appeared sporadically and were only present in one of the two years of study: *Aureobasidium* (*A.*) *pullulans*, *St. bacillaris*, and *K. servazzi*.

Variations in total yeast populations between 2022 and 2023 are much more evident in LFs (Figure 2), those carried out exclusively with yeasts present on grapes. Considering that in both years vineyard management practices have been similar, these data would indicate that inter-annual variations in total yeast populations were more pronounced in LF, indicating that grape microbiota is susceptible to climatic variations during the ripening period, which would affect both the health of the grapes and the composition of the musts, and thus, to the development of different yeast species during fermentation. These results would concur with those of Schütz and Gafner [45], who consider that the population of yeasts can be considered to be dependent on harvest and vineyard.

## 4. Conclusions

The use of indigenous starters, carefully chosen from specific environmental microbiota, is essential for producing wines with region-specific characteristics. Unlocking microbial diversity in spontaneous fermentations represents the pivotal first step in selecting native yeasts for winery-specific wines. From the 1100 yeast colonies identified in this work, *S. cerevisiae* and *H. uvarum* were the most dominant species. Nevertheless, other non-*Saccharomyces* yeasts such as *L. thermotolerans*, *M. pulcherrima*, *St. bacillaris*, and *H. vineae*, all with proven potential as starter cultures, were also detected. The distribution of these species across the 16 fermentations studied was, however, notably heterogeneous. The presence and proportion of yeast species were shaped by numerous factors, with annual climatic conditions, the vineyard’s specific traits, and inoculation from the winery ecosystem exerting greater influence on the microbiota than the grape variety itself.

Therefore, to maximize the recovery of these native yeasts, both non-*Saccharomyces* and *S. cerevisiae*, it is advisable to isolate colonies from vinifications conducted under diverse conditions and across several vintages. This strategy would provide a collection of yeasts that, after oenological evaluation and selection, could serve as invaluable, tailor-made starter cultures for a winery. Additionally, exploring the intraspecific diversity, by studying the different clones within each species across vineyards and vintage, would offer important insights, further enriching the yeast repository for optimized winery- specific applications.

## Figures and Tables

**Table 1:** Degree of maturity (PAS—probable alcoholic strength) of the grapes from the 4 vineyards in the two years studied.

Vineyard	Grape Variety	PAS (%)	
		2022	2023
Vineyard 1	Tempranillo	14.4	15.2
Vineyard 2	Tempranillo	13.5	12.6
Vineyard 3	Graciano	15.3	13.1
Vineyard 4	Tempranillo	15.3	15.5

## Data Availability

The original contributions presented in this study are included in the article. Further inquiries can be directed to the corresponding authors.
